# Patient‐specific psychological characteristics and personality structure affect post‐operative outcomes and readiness to return to sport following medial patellofemoral ligament reconstruction

**DOI:** 10.1002/jeo2.70472

**Published:** 2025-11-16

**Authors:** Lisa Rahn, Andrea Achtnich, Moritz Brunner, Lukas N. Muench, Maximilian Hinz, Julian Mehl, Sebastian Siebenlist, Armin Runer

**Affiliations:** ^1^ Department of Sports Orthopaedics Technical University of Munich Munich Germany; ^2^ Department of Trauma Surgery Regensburg University Medical Center Regensburg Germany; ^3^ Center for Musculoskeletal Surgery Charité – Universitätsmedizin Berlin Berlin Germany

**Keywords:** MPFL, patellofemoral instability, personality structure, psychological characteristics, psychological readiness, return to sport, risk factors

## Abstract

**Purpose:**

The purpose of this study was to evaluate the influence of patient‐specific psychological characteristics and personality structure on the functional outcomes and return to sports (RTS) after isolated medial patellofemoral ligament (MPFL) reconstruction for patellofemoral instability (PFI).

**Methods:**

Patients who underwent isolated MPFL reconstruction for PFI between 2017 and 2020 were retrospectively included. Minimum 18 months post‐operatively, patient‐reported outcome measures, including the Banff Patellofemoral Instability Instrument 2.0 (BPII 2.0), visual analogue scale (VAS) for pain and function, Tegner activity scale (TAS) and Marx activity rating scale (MARS) were evaluated. The MPFL‐Return to Sport after Injury (MPFL‐RSI) scale was used to determine psychological readiness to RTS. Kinesiophobia was measured using the Tampa Scale of Kinesiophobia (TSK), and the tendency to catastrophize pain was measured using the pain catastrophizing scale (PCS). Self‐efficacy was assessed using the General Self‐Efficacy Short Scale‐3 (GSE‐3 scale). Personality structure was classified using a variant of the Big Five Inventory (BFI‐10). The Live Orientation Test (LOT‐R) was used to measure generalized optimism/pessimism. The primary outcome was the correlation between MPFL‐RSI score and BPII 2.0. The data were statistically analyzed using Pearson or Spearman correlation analysis as appropriate.

**Results:**

In total, 54 patients (24 ± 8 years, follow‐up: 35.8 ± 12.8 months) were included. MPFL‐RSI and BPII 2.0 correlated inversely with VAS for pain and function, fear of movement (TSK) and pain catastrophizing (PCS). Both the MPFL‐RSI and BPII 2.0 correlated significantly with self‐efficacy as well as with each other. The MPFL‐RSI correlated inversely with the personality characteristic ‘neuroticism’ (BFI‐10) and positively with sporting activity (MARS).

**Conclusion:**

Individual psychological characteristics and personality structure significantly correlate with the functional outcome and psychological readiness to RTS after MPFL reconstruction. Increased self‐efficacy, reduced pain catastrophizing and exercise phobia are associated with better post‐operative knee function. Preoperative assessment and consideration of these factors may inform tailored prehabilitation.

**Level of Evidence:**

Level IV.

**Abbreviations**
ACL: anterior cruciate ligament
ACT: Acceptance and Commitment Therapies
BFI‐10: Big Five Inventory
BMI: body mass index
BPII 2.0: Banff Patellofemoral Instability Instrument 2.0
GSE‐3: General Self‐Efficacy Short Scale‐3
LOT‐R: Live Orientation Test
MARS: Marx activity rating scale
MPFL: medial patellofemoral ligament
MPFL‐RSI: MPFL‐Return to Sport after Injury
PCS: pain catastrophizing scale
PFI: patellofemoral instability
PROM: patient‐reported outcome measures
TAS: Tegner activity scale
TSK: Tampa Scale of Kinesiophobia
VAS: visual analogue scale

## INTRODUCTION

Patellofemoral instability (PFI) is a multifactorial and potentially severely disabling condition affecting mainly young patients. The clinical presentation of PFI varies, ranging from slight feelings of instability and pain to patellar subluxation and recurrent‐ or habitual dislocations. Frequently, patients suffer from significant limitations in daily activities and sports, making a surgical patellar stabilization necessary. In these patients, when no major osseous patellofemoral risk factors are present, medial patellofemoral ligament (MPFL) reconstruction is considered the gold standard for operative treatment. Despite favourable objective and clinical parameters, the outcomes regarding post‐operative results, return to sport (RTS) and restoration of preoperative performance levels often exhibit heterogeneity [1, 15, 37, 50]. When patients report unsatisfactory post‐operative outcomes, the underlying reasons are not always clearly attributable to objective clinical or radiological findings. Thus, other factors may significantly influence the outcomes in these patients. Recently, psychological factors and patient characteristics have gained increasing importance in explaining both favourable and unfavourable post‐operative results [1, 3, 15, 24, 34, 41, 50]. Key factors affecting satisfaction and RTS include fear of movement (kinesiophobia), an aggravated negative mental state in catastrophizing pain, the patient's belief in their own ability to manage challenging life situations (self‐efficacy), and the extent of their positive (optimism) or negative (pessimism) outlook on life [2, 4, 12, 34]. The purpose of this study was to investigate the influence of patient‐specific psychological characteristics and personality structure on post‐operative outcomes and readiness for RTS following isolated MPFL reconstruction for PFI. To date, there is limited evidence examining the impact of personal psychological factors on outcomes after MPFL reconstruction. It was hypothesized that psychological factors positively correlate with functional scores and RTS scores.

## MATERIALS AND METHODS

The present study was approved by the ethics committee of the Technical University of Munich (2022‐223‐S‐NP) and conducted according to the Declaration of Helsinki. All patients provided written informed consent prior to study inclusion.

All patients over the age of 16 who underwent isolated MPFL reconstruction between January 2017 and December 2020 at a single institution with a minimum follow‐up of 18 months were retrospectively included. The final follow‐up rate was 80.6%, which corresponds to 54 patients with a follow‐up of 35.8 ± 12.8 months (detailed patient enrolment in the Results section). Patients with prior ipsilateral knee surgery, concomitant pathologies or those with patellofemoral risk factors, including high grades of trochlear dysplasia (Dejour ≥ B), patella alta (Caton‐Deschamps index ≥ 1.4), tibial tubercle–trochlear groove index ≥ 20 mm, tibial tubercle–posterior cruciate ligament index ≥ 24 mm, valgus alignment (≥5°) or rotational deformities (anteversion ≥ 25°) were excluded. Furthermore, non‐native German or English speakers were excluded.

### Surgical technique

Preoperative plain knee radiographs and MRI scans were obtained from all patients. Routine arthroscopy was performed before MPFL reconstruction. Indications for MPFL reconstruction were two or more patella dislocations or persistent patella instability despite conservative treatment and a clinically and radiologically confirmed MPFL rupture. MPFL reconstruction was performed using a double‐bundle gracilis tendon (GT) autograft technique. To harvest the GT, a 3‐cm‐long, vertical skin incision was performed medial to the tibial tubercle. The graft was subsequently armed with a 2.0 resorbable suture on both ends. Through a longitudinal skin incision, the medial border of the proximal patella was visualized, and two guidewires were inserted. Subsequently, two 3.5‐mm bone tunnels were drilled at the medial patellar border. Both graft ends were sequentially secured into the patellar holes utilizing a 3.5 SwiveLock (Arthrex). Under fluoroscopic guidance, the anatomical femoral attachment of the MPFL was identified anterior to the posterior femoral cortex, distal to the origin of the medial condyle, and proximal to the most posterior point of Blumensaat's line. The site was marked with a pin and then over‐reamed with a 6 mm drill. The graft was subsequently passed between the joint capsule and the extensor retinaculum, and then pulled into the prepared bone tunnel. Fixation was achieved using a 6 × 20 mm interference screw at 20° of knee flexion, with the lateral border of the patella being flush with the lateral border of the trochlear groove. Care was taken to avoid over‐tensioning the graft during this process.

### Post‐operative rehabilitation

Post‐operatively, partial weight‐bearing (20 kg) for 6 weeks was allowed. The degree of ROM was limited to 90° of flexion for the first 6 weeks. Following check‐up at 6 weeks post‐operatively, full weight‐bearing was encouraged. Physical therapy started on the first post‐operative day with passive flexion and continued two to three times per week thereafter.

### Outcome measurements

All included patients were contacted via e‐mail or phone for personal follow‐up evaluation. Patient‐specific data, including age at the time of surgery, sex and body mass index (BMI), were extracted from the hospital's database.

Patient‐reported outcome measures (PROM) comprised the visual analogue scale (VAS) for pain and function (from 0 = no pain/full function to 10 = maximum pain/complete functional limitation), Banff Patellofemoral Instability Instrument 2.0 score (BPII 2.0) to depict the perceived quality of life of patients with PFI as well as Marx Activity Score (MARS) and Tegner Activity Score (TAS) as an objective parameter for physical activity level [5, 20, 30, 42, 52]. Further, the MPFL‐RSI scale was used to measure psychological readiness to RTS [24, 49]. This scale includes twelve items that assess risk perception, confidence in the knee joint and emotions.

To measure fear of movement, the Tampa Scale of Kinesiophobia (TSK) was utilized [26, 32, 43, 53]. The objective of this score is to assess pain‐related coping behaviours and kinesiophobia through eleven statements. A higher total score indicates greater levels of kinesiophobia. To assess pain catastrophizing, the pain catastrophizing scale was applied [31, 40]. The experience of pain‐related feelings and thoughts is assessed through 13 statements. Respondents indicate on a scale the extent to which these statements reflect their sensations. A higher total score indicates greater levels of catastrophizing.

To assess the participants' sense of self‐efficacy, the General Self‐Efficacy Short Scale (GSE‐3) was used [6, 16].

In this context, general self‐efficacy refers to the confidence in one's ability to master difficult life situations or health‐related challenges through personal competencies. This score is standardized with three questions to assess this concept.

Additionally, the Big Five Inventory (BFI‐10) was queried [36]. The Big Five describe the five dimensions of personality, categorized under the Five‐Factor Model as Extraversion, Neuroticism, Openness, Conscientiousness and Agreeableness [36]. The BFI‐10 is an economical short‐scale consisting of 10 questions designed to measure and assign these dimensions. Research has demonstrated that individuals with varying personality traits exhibit differences in health behaviours and experiences of stress [48, 51].

The German version of the Revised Life Orientation Test (LOT‐R) is implemented to assess the general optimism or pessimism of the respondents [18]. Numerous studies have demonstrated that an optimistic attitude is associated with better physical and psychological well‐being, health behaviours and recovery outcomes [47, 54].

### Statistical analysis

Statistical analysis of the collected data was conducted using IBM SPSS Statistics 20.0 (IBM‐SPSS). Descriptive statistics were calculated for both continuous and categorical variables. Continuous data are reported as mean and standard deviation, while categorical data are presented as percentages. Spearman correlation was utilized to calculate correlations between metric and ordinal data (e.g., MPFL‐RSI and VAS Pain and Function/TSK/TAS/GSE‐3/MARS/LOT‐R Optimism and Pessimism/PCS/BPII 2.0; BPII 2.0 and VAS Pain and Function/TSK/TAS/GSE‐3/MARS/LOT‐R Optimism and Pessimism/PCS/BFI‐10; PCS and TSK/VAS Pain/LOT‐R Optimism and Pessimism/BFI‐10). Pearson correlation coefficient was utilized to calculate correlations between metric data pairs (RSI and BPII 2.0). A *p* value of <0.05 was considered statistically significant. Correlation between factors was classified according to Landis and Koch, who characterized values <0 as indicating no agreement, 0–0.20 as slight, 0.21–0.40 as fair, 0.41–0.60 as moderate, 0.61–0.80 as substantial and 0.81–1 as almost perfect agreement [27].

## RESULTS

**Figure 1:**
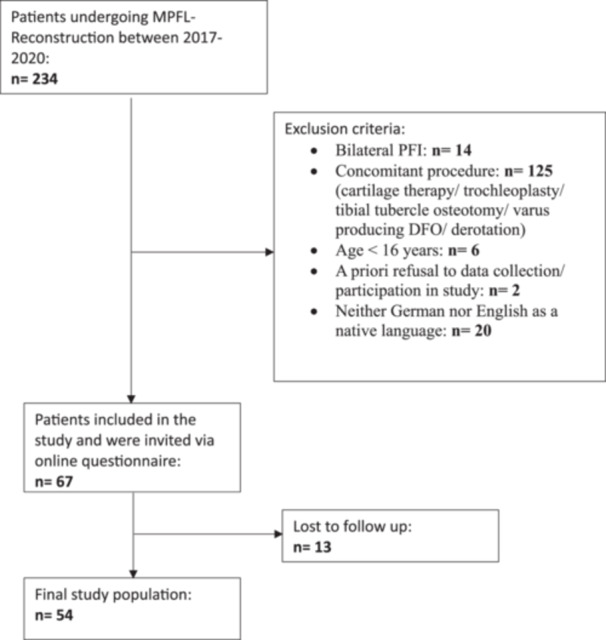
Flowchart of patient enrolment. DFO, distal femoral osteotomy; MPFL, medial patellofemoral ligament; PFI, patellofemoral instability.

In total, 54 patients were included at a mean follow‐up of 35.8 ± 12.8 months. Details on patient enrolment and patient demographics are reported in Figure 1 and Table 1. The final follow‐up rate was 80.6%.

**Table 1:** Patient characteristics.

Variable	Total study group
Number of patients	54 [100]
Age^a^ [years]	23.6 ± 7.4
Male	28 [50.9]
BMI [kg/m^2^]	24.5 ± 4.1
Follow‐up time [months]	35.7 ± 12.8
Patellofemoral risk factors	
Trochlea dysplasia (Dejour)	
No dysplasia	14 [25.5]
Dejour A	18 [32.7]
Dejour B	13 [23.6]
Dejour C	1 [1.8]
Dejour D	0 [0]
Sulcus angle [degree]	148 ± 8
TT‐TG [mm]	12.4 ± 3.9
TT‐PCL [mm]	18.4 ± 6.2
LTI	14.23 ± 4.4
CDI	1.30 ± 0.3
X‐ray of the leg‐axis	
Valgus [*n* = 25, degree]	1.3 ± 0.3
Varus [*n* = 9, degree]	9 ± 1.7

*Note*: Data presented as mean ± SD or *n* [%] if not otherwise stated.

Abbreviations: BMI, body mass index; CDI, Caton–Deschamps Index; LTI, lateral trochlear inclination; TT‐PCL, tibial tuberosity–posterior cruciate ligament distance; TT‐TG, tibial tuberosity–trochlear groove distance.

^a^ Age at time of surgery.

### Outcome measurement

Overall good post‐operative functional results with low pain values were reported (Table 2).

**Table 2:** Outcome measurements.

Score	Mean ± SD
BPII 2.0	73.4 ± 18.1
VAS Pain	2.3 ± 1.5
VAS Function	2.7 ± 1.7
TAS	5.4 ± 1.8
MARS	6.1 ± 4.2
MPFL‐RSI	62.1 ± 24.8
TSK	19.3 ± 5.6
PCS	9.8 ± 10.6
GSE‐3	4.3 ± 0.6
BFI‐10	
Extraversion	3.3 ± 1
Neuroticism	2.9 ± 1
Openness	3.4 ± 1
Conscientiousness	3.8 ± 0.7
Agreeableness	3.5 ± 0.8
LOT‐R Optimism	7.5 ± 2.4
LOT Pessimism	10.0 ± 2.1

Abbreviations: BFI‐10, Big Five Inventory (10 items); BPII 2.0, Banff Patellofemoral Instability Instrument 2.0; GSE‐3, General Self‐Efficacy Short Scale‐3; LOT‐R, Life Orientation Test–Revised (Optimism/Pessimism); MARS, Marx activity rating scale; MPFL‐RSI, MPFL–Return to Sport after Injury; PCS, pain catastrophizing scale; SD, standard deviation; TAS, Tegner activity scale; TSK, Tampa Scale of Kinesiophobia; VAS P/F, visual analogue scale for pain/function.

Correlation between the MPFL‐RSI scale and knee function, pain, sporting activity and psychological profile.

**Figure 2:**
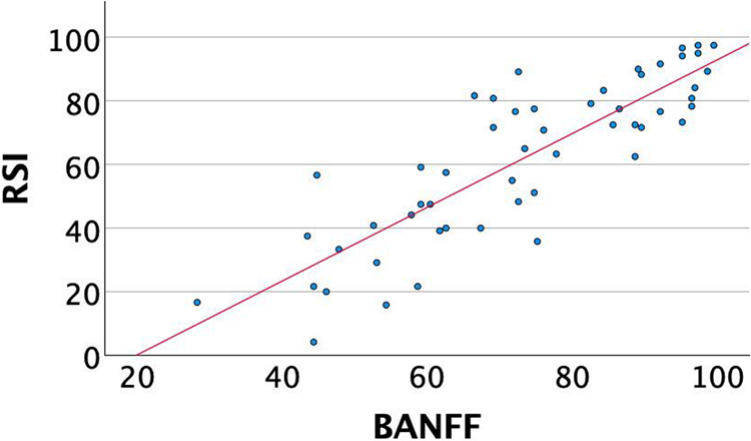
Correlation of MPFL‐RSI and BPII 2.0. BPII 2.0, Banff Patellofemoral Instability Instrument 2.0; MPFL, medial patellofemoral ligament; MPFL‐RSI, MPFL‐Return to Sport after Injury.

**Figure 3:**
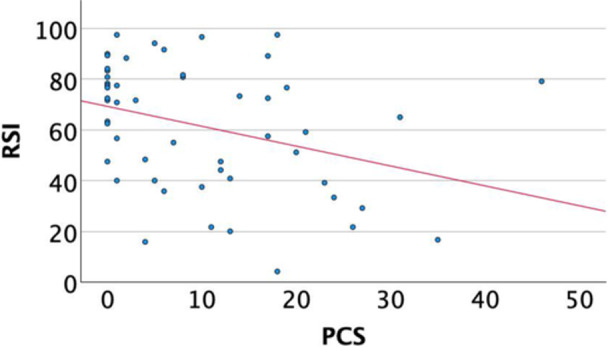
Correlation of MPFL‐RSI and PCS. MPFL, medial patellofemoral ligament; MPFL‐RSI, MPFL‐Return to Sport after Injury; PCS, pain catastrophizing scale.

**Figure 4:**
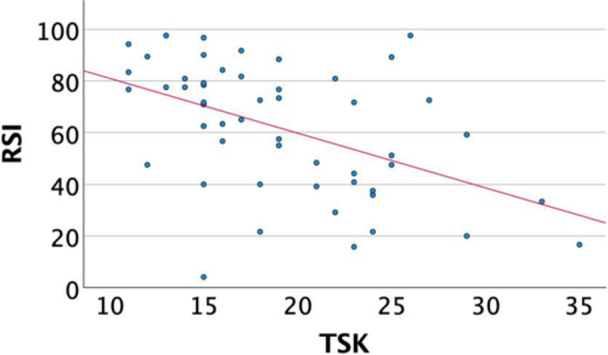
Correlation of MPFL‐RSI and TSK. MPFL, medial patellofemoral ligament; MPFL‐RSI, MPFL‐Return to Sport after Injury; TSK, Tampa Scale of Kinesiophobia.

A significant inverse correlation was revealed between the MPFL‐RSI score and the VAS score for pain (*r* = −0.58, *p* < 0.001) and knee function (*r* = −0.44, *p* < 0.001), fear of movement (*r* = −0.471, *p* < 0.001) and pain catastrophizing (*r* = −0.375, *p* = 0.006). Additionally, the MPFL‐RSI scale correlated significantly with self‐efficacy (r = 0.41, *p* = 0.003), the BPII 2.0 score (*r* = 0.85, *p* < 0.001) and the MARS for athletic activity (*r* = 0.318, *p* = 0.020). A significant negative correlation was shown between the MPFL‐RSI score the personality trait ‘Neuroticism’ (*r* = −0.328, *p* = 0.018) (Table 3) (Figures 2, 3, 4).

**Table 3:** Correlation analysis of MPFL RSI and VAS, TSK, PCS, GSE, BPII 2.0, MARS and BFI‐10.

Scores	*r*	*p*
VAS Pain^S^	−0.581	<0.001
VAS Function^S^	−0.445	<0.001
TSK^S^	−0.471	<0.001
PCS^S^	−0.375	0.006
GSE‐3^S^	0.406	0.003
BPII 2.0^P^	0.851	<0.001
MARS^S^	0.318	0.020
BFI‐10 (Neurocism)^S^	−0.3280	0.018
LOT‐R Optimism^S^	−0.197	0.162
LOT‐R Pessimism^S^	0.154	0.275
TAS^S^	0.299	0.099

Abbreviations: BFI‐10, Big Five Inventory (10 items); BPII 2.0, Banff Patellofemoral Instability Instrument 2.0; GSE‐3, General Self‐Efficacy Short Scale‐3; LOT‐R, Life Orientation Test–Revised (Optimism/Pessimism); MARS, Marx activity rating scale; P, Pearson; PCS, pain catastrophizing scale; S, Spearman; TAS, Tegner activity scale; TSK, Tampa Scale of Kinesiophobia; VAS P&F, visual analogue scale for pain and function.

### Correlation of the BPII 2.0 Score with pain, sporting activity and psychological profile

**Figure 5:**
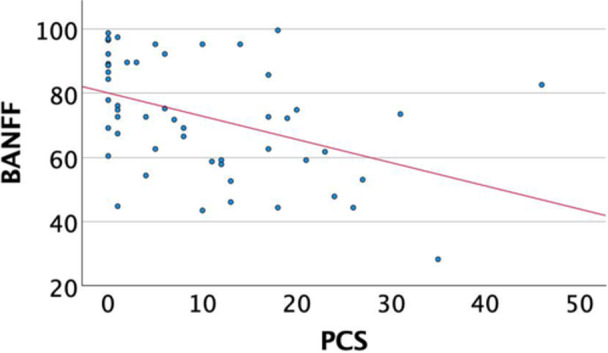
Correlation of BPII 2.0 and PCS. BPII 2.0, Banff Patellofemoral Instability Instrument 2.0; PCS, pain catastrophizing scale.

**Figure 6:**
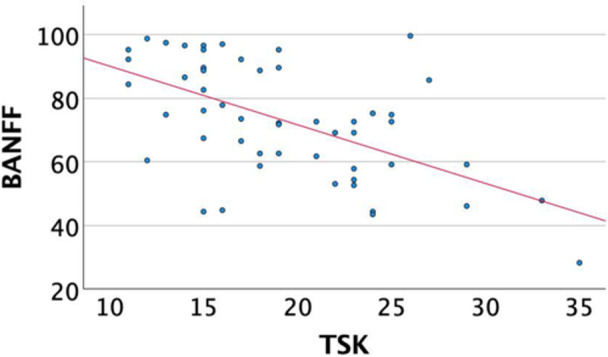
Correlation of BPII 2.0 and TSK. BPII 2.0, Banff Patellofemoral Instability Instrument 2.0; TSK, Tampa Scale of Kinesiophobia.

A significant inverse correlation was revealed between the BPII 2.0 score and the VAS score for pain (*r* = −0.63, *p* < 0.001) and function (*r* = −0.62, *p* < 0.001), fear of movement (*r* = −0.525, *p* < 0.001) and pain catastrophizing (*r* = −0.485, *p* < 0.001) (Table 4). Moreover, the BPII 2.0 score was statistically significantly positively correlated with self‐efficacy (*r* = 0.39, *p* = 0.004) and the MPFL‐RSI score (*r* = 0.85, *p* < 0.001) (Table 4) (Figures 5 and 6).

**Table 4:** Correlation analysis of BPII 2.0 and VAS, TSK, PCS, GSE, BFI‐10 and MARS.

Scores	*r*	*p*
VAS Pain^S^	−0.633	<0.001
VAS Function^S^	−0.617	<0.001
TSK^S^	−0.525	<0.001
PCS^S^	−0.485	<0.001
GSE^S^	0.389	0.004
BFI^S^	−0.375	0.006
MARS^S^	0.201	0.145
Lot‐R Opt^S^	−0.201	0.150
Lot‐R Pess^S^	0.054	0.703
TAS^S^	0.148	0.285

Abbreviations: BFI‐10, Big Five Inventory (10 items); BPII 2.0, Banff Patellofemoral Instability Instrument 2.0; GSE‐3, General Self‐Efficacy Short Scale‐3; LOT‐R, Life Orientation Test–Revised (Optimism/Pessimism); MARS, Marx activity rating scale; P, Pearson Correlation; PCS, pain catastrophizing scale; S, Spearman Correlation; TAS, Tegner activity scale; TSK, Tampa Scale of Kinesiophobia; VAS, visual analogue scale.

### Correlation between the VAS for pain/VAS for function and self‐efficacy, Kinesiophobia, pain catastrophizing, psychological readiness, knee‐specific quality of life/‐function and personal traits

A significant inverse correlation was revealed between the VAS score for pain and the GSE‐3 (*r* = −0.353, *p* = 0.010), PCS (*r* = −0.354, *p* = 0.009), MPFL‐RSI (*r* = −0.581, *p* < 0.001) and BPII2.0 (*r* = −0.633, *p* < 0.001) (Table 4).

A significant correlation was revealed between the VAS score for function and the TSK (*r* = 0.278, *p* = 0.044). Significant inverted correlation was found between VAS score for function and the personality trait ‘Openness’ of the BFI‐10 (*r* = −0.313, *p* = 0.023), MPFL‐RSI (*r* = −0.445, *p* < 0.001) and BPII 2.0 (*r* = −0.617, *p* < 0.001) (Table 5).

**Table 5:** Correlation analysis of VAS for pain and function.

	VAS for pain	
Scores	*r*	*p*
GSE‐3^S^	−0.353	0.010
PCS^S^	−0.354	0.009
TSK^S^	0.375	0.006
MPFL‐RSI^S^	−0.581	<0.001
BPII 2.0^S^	−0.633	<0.001

	VAS for function	
Scores	*r*	*p*
TSK^S^	0.278	0.044
BFI‐10‐Openness^S^	−0.313	0.023
MPFL‐RSI^S^	−0.445	<0.001
BPII 2.0^S^	−0.617	<0.001

Abbreviations: BFI‐10, Big Five Inventory‐10 (Openness); BPII 2.0, Banff Patellofemoral Instability Instrument 2.0; GSE‐3, General Self‐Efficacy Short Scale‐3; MPFL‐RSI, medial patellofemoral ligament–Return to Sport after Injury; PCS, pain catastrophizing scale; S, Spearman correlation.

## DISCUSSION

The main findings of this study were that patient‐specific psychological characteristics and personality structure correlate with patient‐reported post‐operative outcomes, knee‐related quality of life and readiness to RTS in patients who have undergone isolated MPFL reconstruction for PFI. More specifically, the present study showed that psychological readiness to RTS and good knee‐related quality of life are inversely related to knee pain and reduced knee function, kinesiophobia, and pain catastrophizing. Patients with higher pain levels, lower knee function, stronger kinesiophobia and stronger pain catastrophizing achieved worse results. Further, the MPFL‐RSI correlated positively with sports activity.

The results are consistent with previous findings on outcomes after MPFL reconstruction and emphasize the importance of psychological readiness for post‐operative success [24].

However, while prior research highlighted the role of psychological readiness through tools like the MPFL‐RSI and knee‐specific questionnaires, the present study expanded this understanding by correlating these knee‐specific PROMs with validated psychological instruments, including the PCS, GSE‐3 and TSK, to characterize a patient's psychological profile [23]. This approach revealed an association between patient‐specific psychological characteristics, personality structure and functional knee‐related outcomes. Moreover, these findings align with existing literature, demonstrating that in musculoskeletal surgery and chronic pain conditions, factors such as pain catastrophizing and insufficient psychological coping resources can significantly influence post‐operative outcomes and overall patient well‐being [19, 35, 39].

A patient's psychological profile is multifactorial, encompassing a complex interplay of emotional, cognitive and behavioural factors, potentially influencing the post‐operative outcome [11, 14, 51]. An excessive, irrational or debilitating fear of movement due to the belief that it may cause pain, reinjury, or harm (kinesiophobia) plays a decisive role in rehabilitation and return to active life [22]. Similarly, an exaggerated and inappropriate perception of pain that leads to an equally inappropriate response (pain catastrophizing) might lead to slower rehabilitation progress and poorer outcomes [44, 56]. The present data showed a significant relationship between psychological readiness to RTS, post‐operative kinesiophobia and exaggerated pain‐related feelings and thoughts. This is a common finding also in other instability‐related joint pathologies and is regularly associated with poorer post‐operative outcomes [9, 38, 45, 46]. Additionally, pain catastrophizing and kinesiophobia are often interlinked, potentially contributing to less favourable post‐operative outcomes, particularly regarding return to activity and overall patient well‐being [10, 19, 35, 44]. At the same time, it is known that too early, too progressive RTS in relation to persistent knee pain can also be a risk factor for patient dissatisfaction after MPFLR [55].

Building on these insights, further research on MPFL injuries and PFI is essential to deepen our understanding of optimal patient care. Such efforts will help establish standardized preoperative and post‐operative protocols, ensuring more consistent and effective treatment outcomes in the future.

Personality traits have a significant impact on physical and mental health, stress management, coping with challenges and adherence to treatment protocols [21, 25, 41, 51].

This is in line with the results of this study showing that there is a significant association between personality traits, psychological readiness, and knee function in patients after MPFL reconstruction. The present data reveal an inverse correlation between psychological readiness to RTS and the personality trait ‘neuroticism’ of the Big Five personality model. In addition, a significant positive correlation between pain catastrophizing and ‘neuroticism’ was observed, as well as a connection between better function and the personality trait ‘openness’. Health psychology research often relies on the Big Five personality model to explain the influence of psychological characteristics and health, the onset of illness and rehabilitation. It is assumed that personal characteristics or their expression can directly or indirectly influence health behaviour, the onset of illness, and coping mechanisms [8, 48]. The Big Five personality model is based on five stable personality dimensions (Openness, Conscientiousness, Extraversion, Agreeableness and Neuroticism) with certain stable characteristics and tendencies attributed to each of these dimensions [36, 48]. For example, patients with ‘neuroticism’, characterized by emotional instability and heightened sensitivity to stress, may be at a higher risk of a more complicated rehabilitation process, as they are likely to react more sensitively to negative influences. Furthermore, neuroticism is characterized by persistent, excessive attention to non‐objectifiable symptoms and increased sensitivity to negative influences. This could intuitively lead to behaviours of the person that negatively influence rehabilitation than in people with predominantly ‘open’ or ‘conscientious’ personality dimensions. Conversely, openness reflects a willingness to embrace new experiences and adapt to novel solutions, potentially improving outcomes in the rehabilitation process. Individuals with heightened levels of neuroticism may benefit from targeted support during rehabilitation, possibly even in the preoperative phase, whereas patients exhibiting openness are likely to adjust effectively to the new situation.

Innovative therapeutic approaches, such as ‘psychologically informed practice’, show promising results as a possible intervention for this constellation of risk factors. As an interface between physical therapy and elements of psychotherapy or psychological support measures, these approaches could be used in a more targeted way to provide optimal support for high‐risk patient during their rehabilitation [22, 29, 33]. The use of ‘Psychologically Informed Practice’ in the context of ACL injuries has been proposed to address rehabilitation risk factors identified using the ACL RSI scale and TSK. This approach aims to transform dysfunctional behaviours into more effective patterns by incorporating biopsychosocial components [4]. So‐called graduated exposure therapies and ‘Acceptance and Commitment Therapies’ (ACT) have also proven to be helpful in musculoskeletal pain conditions [13]. ACT integrates mindfulness with acceptance of thoughts and feelings, non‐judgmental acknowledgement of them and behavioural adjustment based on cognitive‐behavioural principles. The aim of ACT is to improve inner flexibility and reduce avoidance behaviour [13]. Similarly, parts of the concept of ‘practising mindfulness’ could reduce rumination and pain catastrophizing in relation to pain and movement and thus have a positive effect on the healing process and perception [28]. In order to initiate such an intervention, it could be considered in the future to define preoperative cutoff values of the TSK, PCS or BPII 2.0 explicitly used for intervention indication, which assesses and classifies the risk factors for a delayed rehabilitation process [7].

Components of these therapeutic approaches could also be effective in the rehabilitation of PFI and should be established and further investigated.

Given the strong suggestion in this study that there is a clear relationship between post‐operative function, patient satisfaction, and psychological factors, specific screenings and holistic assessments should be integrated into prehabilitation and rehabilitation to individualize and optimize post‐operative rehabilitation. In summary, while the integration of psychologically informed interventions is partly established in the management of somatic and musculoskeletal conditions, this study highlights a novel approach by integrating standardized orthopaedic and psychological assessment tools. The implementation of a structured and standalized assessment protocol during rehabilitation following stabilizing surgery for PFI appears to be a promising and innovative step towards improving post‐operative outcomes and patient satisfaction. Further studies are needed to assess these parameters pre‐ and postoperatively and to evaluate their impact on outcomes [17, 34].

This study has some limitations. First, as a retrospective study, this work is subject to inherent limitations, including selection bias, recall bias, and potential confounding. Second, by including only patients with isolated MPFL reconstructions without any additional interventions or previous surgeries, the number of patients is small, but the cohort is homogeneous. Third, the questionnaire was completed only post‐operatively, and no preoperative assessment was implemented. Fourth, the small sample size limited more in‐depth statistical analysis. Consequently, it is necessary to consider the potential for a certain degree of false positive results.

## CONCLUSION

Individual psychological characteristics and personality structure show a significant correlation with functional outcomes and psychological readiness to RTS after isolated MPFL reconstruction. Increased self‐efficacy, reduced pain catastrophizing and kinesiophobia are associated with better perceived post‐operative knee function. The preoperative assessment and consideration of these factors may inform patient‐specific therapeutic strategies.

## AUTHOR CONTRIBUTIONS

Lisa Rahn, Andrea Achtnich and Armin Runer designed the study. Lisa Rahn and Moritz Brunner collected data. Lisa Rahn and Armin Runer performed the statistical analysis. Lisa Rahn, Moritz Brunner and Armin Runer wrote the manuscript. Lukas N. Muench, Maximilian Hinz, Sebastian Siebenlist and Armin Runer assisted with data interpretation and critically reviewed the manuscript. All authors read and approved the final manuscript.

## CONFLICT OF INTEREST STATEMENT

Sebastian Siebenlist is a consultant for Arthrex GmbH, KLS Martin Group and medi GmbH & Co. KG. Andrea Achtnich is a consultant for Arthrex GmbH. Julian Mehl is a consultant for Arthrex GmbH and Ormed GmbH. The remaining authors declare no conflicts of interest.

## ETHICS STATEMENT

The study was approved by the Institutional Review Board of the Technical University of Munich (2022‐223‐S‐NP), and the study was performed in accordance with the Declaration of Helsinki. Written informed consent was obtained from all patients.

## Data Availability

The data that support the findings of this study are available from the corresponding author upon reasonable request.
